# The association between interferon lambda 3 and 4 gene single-nucleotide polymorphisms and the recovery of COVID-19 patients

**DOI:** 10.1186/s12985-021-01692-z

**Published:** 2021-11-14

**Authors:** Pooneh Rahimi, Rahil Tarharoudi, Alireza Rahimpour, Jalal Mosayebi Amroabadi, Iraj Ahmadi, Enayat Anvari, Seyed Davar Siadat, Mohammadreza Aghasadeghi, Abolfazl Fateh

**Affiliations:** 1 Hepatitis and AIDS Department, Pasteur Institute of Iran, Tehran, Iran; 2 Viral Vaccine Research Center, Pasteur Institute of Iran, Tehran, Iran; 3 Department of Molecular and Cellular Biology, Faculty of Advanced Science and Technology, Tehran Medical Sciences, Islamic Azad University, Tehran, Iran; 4 Department of Biology, Science and Research Branch, Islamic Azad University, Tehran, Iran; 5 Artificial Intelligence and Multi-Omics Center (AIMOC), Stavanger, Norway; 6 Department of Physiology, School of Medicine, Ilam University of Medical Science, Ilam, Iran; 7 Department of Mycobacteriology and Pulmonary Research, Pasteur Institute of Iran, Tehran, Iran; 8 Microbiology Research Center (MRC), Pasteur Institute of Iran, Tehran, Iran

**Keywords:** SARS-CoV-2, COVID-19, Interferon lambda 3, Interferon lambda 4, Single-nucleotide polymorphisms

## Abstract

**Background:**

The recent pandemic caused by severe acute respiratory syndrome coronavirus-2 (SARS-CoV-2) has elevated several clinical and scientific questions. These include how host genetic factors influence the pathogenesis and disease susceptibility. Therefore, the aim of this study was to evaluate the impact of interferon lambda 3 and 4 (*IFNL3/4*) gene polymorphisms and clinical parameters on the resistance and susceptibility to coronavirus disease 2019 (COVID-19) infection.

**Methods:**

A total of 750 SARS-CoV-2 positive patients (375 survivors and 375 nonsurvivors) were included in this study. All single-nucleotide polymorphisms (SNPs) on *IFNL3* (rs12979860, rs8099917, and rs12980275) and *IFNL4* rs368234815 were genotyped by the polymerase chain reaction-restriction fragment length polymorphism (PCR–RFLP) method.

**Results:**

In this study, a higher viral load (low PCR Ct value) was shown in nonsurvivor patients. In survivor patients, the frequency of the favorable genotypes of *IFNL3/4* SNPs (rs12979860 CC, rs12980275 AA, rs8099917 TT, and rs368234815 TT/TT) was significantly higher than in nonsurvivor patients. Multivariate logistic regression analysis has shown that a higher low-density lipoprotein (LDL), erythrocyte sedimentation rate (ESR), C-reactive protein (CRP), and PCR Ct value, and lower 25-hydroxyvitamin D, and also *IFNL3* rs12979860 TT, *IFNL3* rs8099917 GG, *IFNL3* rs12980275 GG, and *IFNL4* rs368234815 ∆G/∆G genotypes were associated with the severity of COVID-19 infection.

**Conclusions:**

The results of this study proved that the severity of COVID-19 infection was associated with clinical parameters and unfavorable genotypes of *IFNL3/IFNL4* SNPs. Further studies in different parts of the world are needed to show the relationship between severity of COVID-19 infection and host genetic factors**.**

## Introduction

In December 2019, a new coronavirus known as severe acute respiratory syndrome coronavirus-2 (SARS-CoV-2) emerged from Wuhan, China, which causes coronavirus disease 2019 (COVID-19). This virus is not like the other coronaviruses that are commonly found in humans and cause common cold like symptoms. COVID-19 is known as an acute respiratory infectious disease that is mostly transmitted via the respiratory tract [1]. Although the COVID-19 pathology has not yet been completely understood, the virus can cause a wide spectrum of symptoms, ranging from asymptomatic through mild symptoms of upper respiratory tract infection to life-threatening conditions [2]. The rate of mortality varies by location. However, it is difficult to assess the mortality of the case caused by the disease until the pandemic is over, and according to a study, in different regions, the number may vary from 3.75 to 13% of the total population [3].

Host genetic factors could play a vital role in determining the clinical manifestations and infection outcome. Recently, a feasible role of single-nucleotide polymorphisms (SNPs) involved in the host antiviral responses against SARS-CoV-2 has been suggested, and there is increasing evidence that some SNPs could influence the COVID-19 course susceptibility and severity [4]. Family clusters of severe cases have been indicated around the world, supporting further efforts to discover these genetic factors to better prepare for future waves [5, 6].

Several studies have shown that some SNPs on interferon lambda type 3 and 4 (*IFNL3/4*) signaling are involved in the sustained antiviral response and the immunity regulation [7–9]. The homozygote variants of *IFNL3/4* gene could be correlated with a viral clearance decrease in children affected by acute respiratory infections [10]. Several reports have shown that these SNPs on *IFNL3/4* gene were able to restrict the replication of viruses such as respiratory syncytial virus, Zika virus, severe acute respiratory syndrome coronavirus, human metapneumovirus, vaccinia virus, influenza A/V viruses, herpes simplex virus-2, and others in mice [11–13].

In Iran, no study has evaluated the impact of SNPs on *IFNL3/4* gene in COVID-19 patients. Therefore, the purpose of the current study was to investigate the association of three important *IFNL3* SNPs (rs12979860, rs8099917, and rs12980275) and *IFNL4* rs368234815 with the resistance and susceptibility to COVID-19 infection.

## Materials and methods

### Study population

The present study was performed at the Pasteur Institute of Iran (PII) from March 2020 to September 2020. A total of 750 patients with COVID-19 infection were selected. Reverse transcriptase real-time polymerase chain reaction (rtReal Time-PCR) of oropharyngeal or nasopharyngeal swab samples was used for the detection of SARS-CoV-2 infection. The patients did not have any underlying medical conditions, including heart and chronic kidney disease, diabetes, obesity, liver disease, cancer, human immunodeficiency virus (HIV), pregnancy, chronic obstructive pulmonary disease, cystic fibrosis, asthma (moderate-to-severe), and etc.

Approximately 10 mL of blood samples were obtained from positive patients. Peripheral blood mononuclear cells (PBMCs) were extracted through Ficoll (Ficoll-Paque PLUS, GE Healthcare, USA) density gradient centrifugation and stored at − 20 °C. The laboratory parameters including Alanine aminotransferase (ALT), aspartate aminotransferase (AST), alkaline phosphatase (ALP), fasting blood glucose (FBS), low-density lipoprotein (LDL), cholesterol, triglyceride (TG), high density lipoprotein (HDL), blood urea nitrogen (BUN), serum creatinine, uric acid, 25-hydroxyvitamin D, C-reactive protein (CRP), white blood cells (WBC), hemoglobin, erythrocyte sedimentation rate (ESR), triiodothyronine (T3), thyroxine (T4), thyroid-stimulating hormone (TSH), platelets, and real-time PCR Ct values were extracted from the patients’ records.

### DNA extraction and *IFNL3* and *IFNL4* SNPs genotyping

The DNA of patients with COVID-19 infection was extracted using the High Pure PCR Template Preparation Kit (Roche Diagnostics Deutschland GmbH, Mannheim, Germany), according to the manufacturer’s instructions. The *IFNL3* SNPs (rs12979860, rs8099917, and rs12980275) and *IFNL4* rs368234815 were genotyped using the PCR-based restriction fragment length polymorphism assay, as previously described [14, 15]. Briefly, for *IFNL3* rs12979860, the primers were: 5′- CTCTGCACAGTCTGGGATTCCT-3′ (forward), and 5′-CTGAGGGACCGCTACGTAAGTC-3′ (reverse). For *IFNL3* rs12980275, the primer sequences were: 5′-GAGAGCAAGAGGAGGGAAGGAA-3′ (forward), and 5′-GTGTGCCATTAGCCAGTCAGAT-3′ (reverse). For *IFNL3* rs8099917, the primers were: 5′-TTCACCATCCTCCTCTCATCCCTCAT-3′ (reverse) and 5′-TCCTAAATTGACGGGCCATCTGTTTC-3′ (reverse). For *IFNL4* rs368234815 the primers were: 5′-GACGCAGGACCCCTTGGGACAGGA-3′ (forward) and 5′-TCTGGGCCGCAGTGGCCGCGAGG-3′ (reverse). The PCR product for *IFNL3* rs12979860, rs12980275, rs8099917, *IFNL4* and rs368234815 was of 403, 441, 401, and 227 base pairs, respectively. For the RFLP assay for the *IFNL3* rs12979860, rs12980275, rs8099917, *IFNL4* and rs368234815 was used of the *Bsh1236I*, *Bsl I*, *Mae* *III*, and *MspA1I* restriction endonuclease enzymes, respectively.

### Statistical analysis

A Shapiro–Wilk test was applied for assessing the data normality of continuous variables. Pearson’s chi-square and Mann–Whitney U tests were also used for the evaluation of quantitative variables and continuous variables, respectively. To analyze the correlation of risk factors for COVID-19 resistance and susceptibility, multivariate logistic regression analysis was carried out using the Hosmer–Lemeshow test. Two-tailed *P-*value > 0.05 was considered statistically significant. The area under the receiver-operating characteristic curve (AUC-ROC) analysis was used to evaluate the impact of *IFNL3/4* SNPs in relation to resistance and susceptibility to COVID-19. All data were analyzed using IBM SPSS for windows version 22.0 statistical software (SPSS. Inc., Chicago, IL, USA).

## Results

### Baseline characteristics of COVID-19 patients

This study included 750 patients with COVID-19 infection, who were divided into two groups: survivor (n = 375) and nonsurvivor (n = 375). The laboratory and clinical properties of patients are presented in Table 1. Briefly, the mean age of survivor and nonsurvivor patients was 50.6 ± 11.8 and 59.1 ± 11.9 years, respectively. The susceptibility to COVID-19 infection were significantly associated with high cholesterol (*P* = 0.005), ESR (*P* < 0.001), LDL (*P* = 0.002), CRP (*P* < 0.001) levels, and low 25-hydroxyvitamin D (*P* < 0.001). In this study, a low PCR Ct value was shown in nonsurvivor patients (*P* = 0.028).

**Table 1:** Comparison laboratory parameters between survivors and nonsurvivors patients infected with COVID-19

Variables	Survivors (n = 375)	Nonsurvivors (n = 375)	*P*-value
Mean age ± SD	50.6 ± 11.8	59.1 ± 11.9	0.537
Gender (male/female)	214/161 (57.1/42.9%)	223/152 (59.6/40.4%)	0.098
ALT, IU/L (mean ± SD) (Reference range: 5–40)	32.6 ± 14.8	31.1 ± 23.7	0.326
AST, IU/L (mean ± SD) (Reference range: 5–40)	33.9 ± 15.2	31.2 ± 17.7	0.226
ALP, IU/L (mean ± SD) (Reference range: up to 306)	177.5 ± 94.7	165.7 ± 79.8	0.332
Cholesterol, mg/dL (mean ± SD) (Reference range: 50–200)	115.5 ± 40.2	127.9 ± 49.1	0.005*
TG, mg/dL (mean ± SD) (Reference range: 60–165)	129.1 ± 47.6	136.8 ± 67.1	0.172
LDL, mg/dL (mean ± SD) (Reference range: up to 150)	61.5 ± 29.1	73.3 ± 21.2	0.002*
HDL, mg/dL (mean ± SD) (Reference range: > 40)	32.5 ± 12.9	32.1 ± 11.8	0.866
WBC, 10^9^/L (mean ± SD) (Reference range: 4000–10,000)	7615.4 ± 2641.2	7812.3 ± 2878.1	0.440
CRP, mg/L (mean ± SD) (Reference range: < 10 mg/L Negative)	54.9 ± 20.1	68.4 ± 20.7	< 0.001*
ESR, mm/1st h (mean ± SD) (Reference range: 0–15)	45.8 ± 15.5	56.1 ± 15.4	< 0.001*
FBS, mg/dL (mean ± SD) (Reference range: 70–100)	107.3 ± 41.9	106.1 ± 40.5	0.877
Platelets × 1000/cumm (mean ± SD) (Reference range: 140,000–400,000)	183 ± 67	185 ± 78	0.540
T3, ng/dL (mean ± SD) (Reference range: 2.3–4.2)	3.1 ± 1.6	2.9 ± 1.7	0.181
T4, mcg/dL (mean ± SD) (Reference range: 5.6–13.7)	8.4 ± 6.1	8.9 ± 6.3	0.310
TSH, mu/L (mean ± SD) (Reference range: 0.4–4.5)	3.2 ± 1.9	3.5 ± 2.1	0.651
Hemoglobin, g/dL (mean ± SD) (Reference range: 12–18)	12.5 ± 1.3	13.1 ± 1.7	0.603
BUN, mg/dL (mean ± SD) (Reference range: 15–45)	38.2 ± 7.9	46.2 ± 8.2	0.248
Creatinine, mg/dL (mean ± SD) (Reference range: 0.6–1.4)	1.4 ± 0.5	1.5 ± 0.7	0.219
25-hydroxy vitamin D, ng/mL (mean ± SD) (Sufficiency: 21–150)	35.7 ± 13.5	20.1 ± 9.3	< 0.001*
Real-time PCR Ct values	28.4 ± 10.2	18.4 ± 7.4	0.028*

ALT, alanine aminotransferase; AST, aspartate aminotransferase; ALP, alkaline phosphatase; TG, triglyceride; LDL, low density lipoprotein; HDL, high density lipoprotein; WBC, white blood cells; CRP, C-reactive protein; ESR, erythrocyte sedimentation rate; FBS, fasting blood glucose; T3, triiodothyronine; T4, thyroxine; TSH, Thyroid-stimulating hormone; BUN, Blood urea nitrogen; Ct, cycle threshold; SD, standard deviation. *Statistically significant (< 0.05)

### Association between *IFNL3/4* SNPs and the resistance and susceptibility to COVID-19 infection

**Fig. 1:**
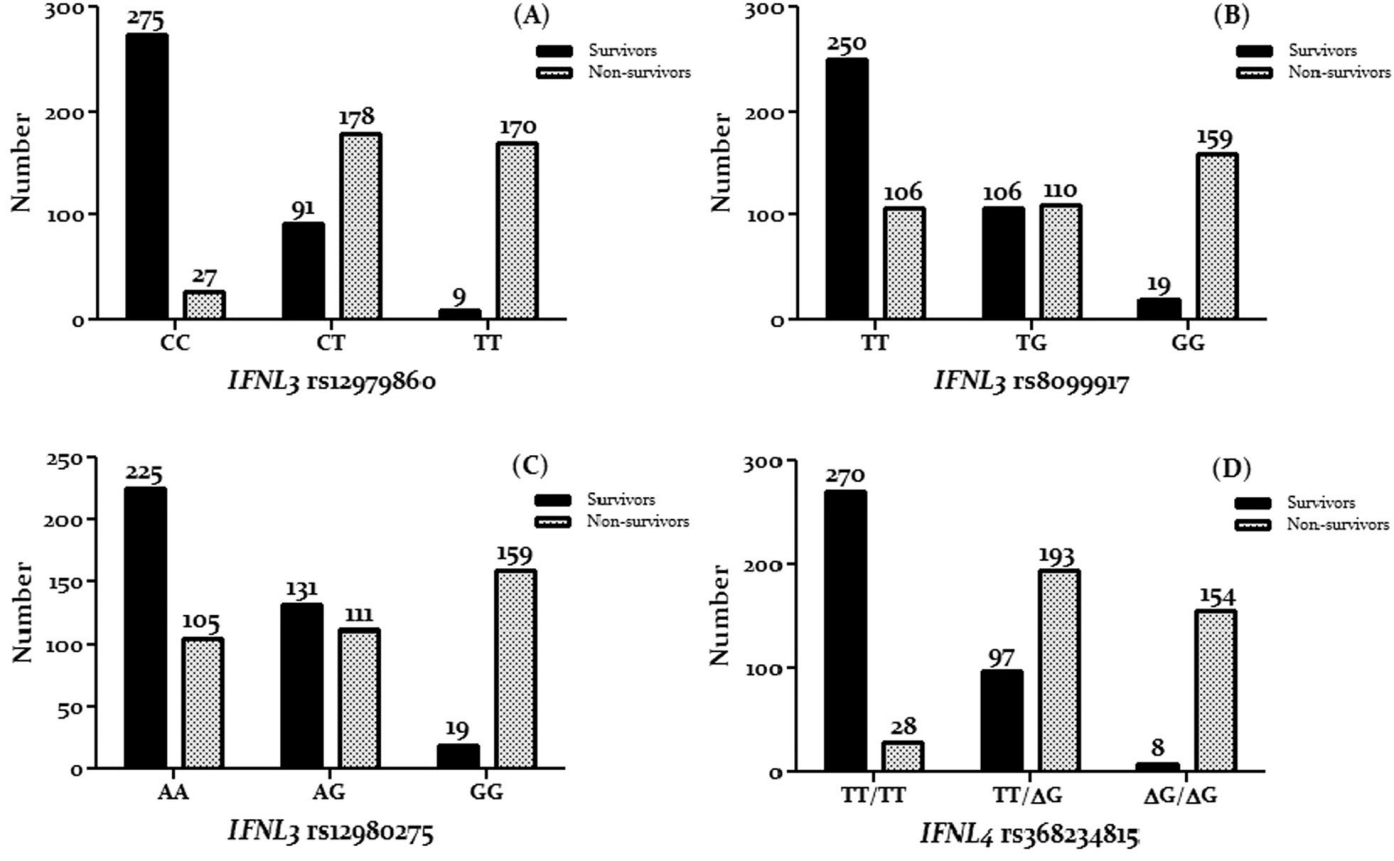
Frequency of *IFNL3* rs12979860 (**A**), rs8099917 (**B**), rs12980275 (**C**), and *IFNL4* rs368234815 (**D**) in COVID-19 patients

The frequency of the favorable genotypes of *IFNL3/4* SNPs (*IFNL3* rs12979860 CC, rs12980275 AA, rs8099917 TT, and *IFNL4* rs368234815 TT/TT) was significantly higher among COVID-19 survivor patients, whereas unfavorable genotypes of *IFNL3/4* (*IFNL3* rs12979860 TT, rs12980275 GG, rs8099917 GG, and *IFNL4* rs368234815 ∆G/∆G genotypes) were observed in COVID-19 nonsurvivor patients (Fig. 1). The result of this study indicated that the patients with a co-expression of the favorable genotypes (*IFNL3* rs12979860 CC, rs8099917 TT, rs12980275 AA, and *IFNL4* rs368234815) had demonstrated a better response to COVID-19 infection compared to patients having other genotypes. Out of the 375 patients who recovered, 225 (60.0%) patients had all favorable genotypes. Out of the 375 patients who died, 153 (40.8%) patients had unfavorable genotypes.

**Fig. 2:**
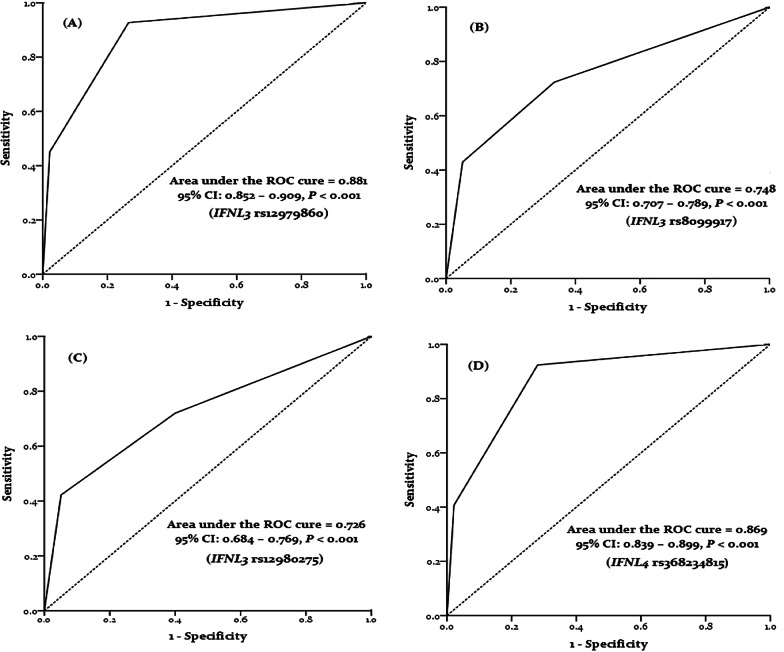
ROC curve with the *IFNL3* rs12979860 (**A**), rs8099917 (**B**), rs12980275 (**C**), and *IFNL4* rs368234815 (**D**) for prediction the mortality rate in COVID-19 patients

The AUC-ROC was 0.881 for *IFNL3* rs12979860, 0.748 for *IFNL3* rs8099917, 0.726 for *IFNL3* rs12980275, and 0.869 for *IFNL4* rs368234815, suggesting that host genetic factors are generally important for the resolution of viral infection (Fig. 2).

### Factors associated with resistance and susceptibility to COVID-19 infection

Using multivariate logistic regression analysis, we evaluated the factors correlated with resistance and susceptibility to COVID-19 infection. In survivor patients, the resistance to COVID-19 infection was associated with LDL (*P* < 0.001), ESR (*P* < 0.001), CRP (*P* = 0.014), 25-hydroxyvitamin D (*P* = 0.032), Real-time PCR Ct values (*P* = 0.045), *IFNL3* rs12979860 CC (*P* < 0.001), *IFNL3* rs8099917 GG (*P* < 0.001), *IFNL3* rs12980275 AA (*P* < 0.001), and *IFNL4* rs368234815 TT/TT (*P* < 0.001) (Table 2).

**Table 2:** Factors associated with survivor patients infected with COVID-19

Factors		
Baseline predictors	OR (95% CI)	*P*-value
LDL (mg/dL)	1.036 (1.017–1.056)	< 0.001*
25-Hydroxyvitamin D, (ng/Ml)	1.029 (1.002–1.056)	0.032*
ESR, (mm/1st h)	0.958 (0.937–0.979)	< 0.001*
CRP, (mg/L)	0.981 (0.966–0.996)	0.014*
Real-time PCR Ct values	0.322 (0.301–0.689)	0.045*
*IFNL3* rs12979860 (CC)	0.742 (0.038–0.943)	< 0.001*
*IFNL3* rs8099917 (TT)	0.294 (0.175–0.494)	< 0.001*
*IFNL3* rs12980275 (AA)	0.416 (0.257–0.675)	< 0.001*
*IFNL4* rs368234815 (TT/TT)	0.122 (0.066–0.224)	< 0.001*

LDL, low density lipoprotein; CRP, C-reactive protein; ESR, erythrocyte sedimentation rate; Ct, cycle threshold; *IFNL3*, interferon lambda 3; *IFNL4*, interferon lambda 4; SD, standard deviation; *Statistically significant (< 0.05)

## Discussion

The present study comprehensively investigated the association between resistance and susceptibility to COVID-19 infection with host genetic variants and clinical parameters. Better management of COVID-19 requires a better understanding of the direct and indirect damage caused by SARS-CoV-2 present in the host [2]. Factors related to viruses (such as viral load) and hosts (SNPs) are associated with diverse clinical outcomes [7].

In this study, a low Ct for rtRT-PCR was indicated in nonsurvivor COVID-19 patients. Several studies have shown that a low PCR Ct value was associated with a higher risk of hospitalization in the intensive care unit (ICU) and, finally, death. It has suggested a correlation between viral loads determined by PCR Ct measurement and disease severity. However, these results need further investigation [2, 16, 17]. As is previously well described [18, 19], in our study, the correlation between severity of COVID-19 and gender (male) was shown. Several investigators have considered the role of potential gender-specific mechanisms modulating the disease progression, including the expression of some genes involved in the regulation of hormone secretion, sex-hormone-driven adaptive and innate immune responses, and aging of the immune system [18].

According to previous reports [20, 21], we found that the deficiency of 25-hydroxy vitamin D is much more prevalent in severe COVID–19 patients requiring admission to ICU, and as a result, the risk of death increases. 25-hydroxy vitamin D plays an important role in the modulation of the immune system, which interferes with the immune system’s cells, including B-/T-cells, macrophages, dendritic cells, and neutrophils [22]. Also, this vitamin inhibits proinflammatory cytokine production and boosts the anti-inflammatory cytokine production [23]. A study has indicated the positive effect of using calcitriol as a vitamin D agonist on Lipopolysaccharides (LPS)-induced acute lung damage in rats. It turned out that the permeability of LPS-induced lung was significantly improved by calcitriol pretreatment. Calcitriol could modulate the expression of several genes, such as angiotensin I-converting enzymes (ACE and ACE2). Interestingly, the ACE receptor is necessary for SARS-CoV-2 to enter the cell [24].

The level of cholesterol, LDL, ESR, and CRP in non-survivors patients was higher than survivor’s patients. In a meta-analysis involving 21 studies indicated that inflammatory biomarkers including ESR and CRP were significantly increased in patients with both survivors and nonsurvivors COVID-19. This increase can be due to high inflammation in these patients. Also, higher membrane cholesterol could promote the entry of SARS-CoV-2 with two important mechanisms including increases furin availability and facilitates greater ACE2 availability. As a result, cholesterol medications may reduce the severity of COVID19 patients [25, 26].

There is little data on the effect of host genetics on SARS-CoV-2 infection and their clinical manifestations. Although a lot of studies have focused their research on the receptors of the virus and several genetic linkages between ACE2 genetic variants and the increased patient susceptibility to the infection, there is limited information on other genes involved in the pathology of the disease [27]. This evidence is consistent with the IFNL’s key role in responding to viral infections sustained by DNA viruses, double-stranded RNA viruses, and positive- and negative-sense RNA viruses. That SNPs in *IFNL* genes were strongly associated with viral infection outcomes stands as proof of the key role type III IFNs play in immune response regulation [28].

Based on the important roles played by *IFNL3/4* SNPs in restricting viral replications, we postulated that these SNPs might be associated with resistance and susceptibility to COVID-19 infection. To the best of our knowledge, we found that patients who simultaneously express favorable genotypes of these SNPs (rs12979860 CC, rs12980275 AA, rs8099917 TT, and rs368234815 TT/TT) showed a better chance of resistance to COVID-19 infection compared to those with unfavorable genotypes. In the former, co-expression of *IFNL3* rs12979860 CC and *IFNL4* rs368234815 TT/TT was a strong predictor for resistance to COVID-19 infection. Amodio el al., demonstrated that individuals that who had unfavorable genotypes of *IFNL3* of rs1297860 (TT) and *IFNL4* rs368234815 ∆G/∆G indicated a less ability in clearance of virus [2].

It has been reported that there is a high linkage disequilibrium between the genotypes rs1297860(C/T) and *IFNL4* rs368234815 (TT/ΔG), which is the cause of the strongest host factor linked to viral clearance. Also, the unfavorable genotypes of *IFNL3-IFNL4* variants, which were found in people of African descent, are correlated with a decrease in viral clearance in children with acute respiratory infections, such as coronavirus and rhinovirus infections [13, 28]. Recently, several studies have shown that in a group of patients with life-threatening COVID-19 pneumonia, patients with inheritable errors of type I IFNs were characterized by neutralizing autoantibodies against type I IFNs [29, 30]. These findings could propose the protective role of type I IFNs signaling against severe SARS-CoV-2 infection [2].

Based on all evidence, the uses of IFNLs as drugs against viral infection has been proposed in COVID-19 patients or in individuals at high risk for infection, and current randomized clinical trials are designed with peg-IFNL1 in the case of acute COVID-19 [31].

The one limitation of this study was the PCR Ct values, although this correlation is not yet well standardized and quantified and, therefore, could be influenced by a low degree of accuracy and precision.

## Conclusions

The present study showed a strong relationship between ESR, CRP, real-time PCR Ct values, 25-hydroxyvitamin D, and *IFNL3/4* SNPs and the severity of COVID-19 infection. We also demonstrated that patients with unfavorable *IFNL3* SNPs and *IFNL4* rs368234815 genotypes were exposed to more severe COVID-19 infection compared to patients with other genotypes.

## Data Availability

All data generated or analyzed during this study are included in this published article.

## Abbreviations

SARS-CoV-2: Severe acute respiratory syndrome coronavirus-2
*IFNL3/4*: Interferon lambda 3 and 4
COVID-19: Coronavirus disease 2019
SNPs: Single-nucleotide polymorphisms
PCR–RFLP: Polymerase chain reaction-restriction fragment length polymorphism
LDL: Low-density lipoprotein
ESR: Erythrocyte sedimentation rate
CRP: C-reactive protein
rtReal Time-PCR: Real-time polymerase chain reaction
HIV: Human immunodeficiency virus
PBMCs: Peripheral blood mononuclear cells
AUC-ROC: Area under the receiver-operating characteristic curve
ICU: Intensive care unit
ACE: Angiotensin I-converting enzymes
